# Combining Antiangiogenic Therapy with Adoptive Cell Immunotherapy Exerts Better Antitumor Effects in Non-Small Cell Lung Cancer Models

**DOI:** 10.1371/journal.pone.0065757

**Published:** 2013-06-14

**Authors:** Shujing Shi, Rui Wang, Yitian Chen, Haizhu Song, Longbang Chen, Guichun Huang

**Affiliations:** Medical Oncology Department of Jinling Hospital, Medical school of Nanjing University, Nanjing, People’s Republic of China; Wayne State University, United States of America

## Abstract

**Introduction:**

Cytokine-induced killer cells (CIK cells) are a heterogeneous subset of ex-vivo expanded T lymphocytes which are characterized with a MHC-unrestricted tumor-killing activity and a mixed T-NK phenotype. Adoptive CIK cells transfer, one of the adoptive immunotherapy represents a promising nontoxic anticancer therapy. However, in clinical studies, the therapeutic activity of adoptive CIK cells transfer is not as efficient as anticipated. Possible explanations are that abnormal tumor vasculature and hypoxic tumor microenvironment could impede the infiltration and efficacy of lymphocytes. We hypothesized that antiangiogenesis therapy could improve the antitumor activity of CIK cells by normalizing tumor vasculature and modulating hypoxic tumor microenvironment.

**Methods:**

We combined recombinant human endostatin (rh-endostatin) and CIK cells in the treatment of lung carcinoma murine models. Intravital microscopy, dynamic contrast enhanced magnetic resonance imaging, immunohistochemistry, and flow cytometry were used to investigate the tumor vasculature and hypoxic microenvironment as well as the infiltration of immune cells.

**Results:**

Our results indicated that rh-endostatin synergized with adoptive CIK cells transfer to inhibit the growth of lung carcinoma. We found that rh-endostatin normalized tumor vasculature and reduced hypoxic area in the tumor microenvironment. Hypoxia significantly inhibited the proliferation, cytotoxicity and migration of CIK cells in vitro and impeded the homing of CIK cells into tumor parenchyma ex vivo. Furthermore, we found that treatment with rh-endostatin significantly increased the homing of CIK cells and decreased the accumulation of suppressive immune cells in the tumor tissue. In addition, combination therapy produced higher level of tumor-infiltration lymphocytes compared with other treatments.

**Conclusions:**

Our results demonstrate that rh-endostatin improves the therapeutic effect of adoptive CIK cells therapy against lung carcinomas and unmask the mechanisms of the synergistic antitumor efficacy, providing a new rationale for combining antiangiogenesis therapy with immunotherapy in the treatment of lung cancer.

## Introduction

Lung cancer is one of leading causes of cancer-related death [1]. Most lung cancer patients are diagnosed at advanced stages (III or IV) and various treatments have emerged including chemotherapy, radiotherapy, target therapy and immunotherapy. CIK cells are heterogeneous cell populations derived from human peripheral blood or mice spleen after in vitro expansion with interferon-γ, interleukin-2 and anti-CD3 antibodies [2]. CIK cells mediate potent MHC-unrestricted cytotoxicity against a variety of tumor cells and can recognize and kill tumor cells without prior exposure or priming. There are two main subpopulations can be distinguished within the bulk culture of in vitro expanded CIK cells, one co-expressing the CD3 and CD56 molecules (CD3^+^CD56^+^) while the other presenting a CD3^+^CD56^−^ phenotype. The antitumor activity of CIK cells has been reported to be mainly restricted to the CD3^+^CD56^+^ cells [3]. Adoptive CIK cells transfer, one of the adoptive immunotherapy represents a promising nontoxic anticancer therapy in the treatment of solid tumors refractory to conventional therapies. However in clinical studies, the therapeutic activity of CIK cells transfer is not as efficient as anticipated [4]. Effective adoptive cell transfer is facing numerous challenges such as systemic immune tolerance and tumor local immune escape. The homing of immune cells to the tumor site is reduced and the antitumor immune functions are inhibited by tumor microenvironment and immunomodulatory properties of suppressive cell populations [5], [6]. Thus it is urgent to find an effective therapy to enhance the adoptive cell transfer efficacy so as to improve clinical effect of cancer patients.

It has been proposed that the effectiveness of cell-based immunotherapies could be affected by the integrity of the tumor vasculature and immunosuppressive tumor microenvironment [7]. Hypoxia is a hallmark of abnormal metabolic environment in solid tumors. Except for being involved in decreased radiation sensitivity and resistance to chemotherapy, hypoxia has emerged as a significant factor of immune tolerance in the tumor microenvironment [8], [9], [10]. Several lines of evidence suggested that antiangiogenesis transiently normalized tumor vasculature, decreased intra-tumor hypoxia and increased lymphocyte infiltration into tumor, which provided a rationale for combining antiangiogenesis therapy with adoptive cell immunotherapy [11], [12]. Antiangiogenesis therapy has been reported to increase the antitumor efficacy of chemotherapy, radiotherapy and immunotherapy in both animal models and in the human [13], [14], [15], [16]. Endostatin is a 20-kDa fragment of type XVIII collagen, capable of reducing the proliferation, migration and invasion of endothelial cells by interacting αvβ1, -αvβ3, and αvβ5 intergrins on the surface of Human umbilical vein endothelial cells (HUVECs) [17]. In phase I and phase II clinical trials in America, endostatin showed minor to none antitumor response although no adverse side effects were found. Rh-endostatin used in the present study is a modified form of endostatin with an additional nine-amino acid sequence which formed another his-tag structure and has been approved for clinical use by the State Food and Drug Administration in China for the treatment of advanced non-small-cell lung cancer in 2005 [18]. It has been proved that rh-endostatin could improve patients’ progress free survival in advanced non-small cell lung cancer (NSCLC) with combination of chemotherapy in randomized trials. We have previously showed that rh-endostatin could improve the antitumor efficacy of paclitaxel in the treatment of lung carcinoma [19]. Rh-endostatin has been reported to enhance the radioresponse for human carcinoma by improving the hypoxic tumor microenvironment [8], [20]. However, until recently, there is little information available in literature about the antitumor effect of combining rh-endostatin with adoptive cell immunotherapy. We conducted this research to determine whether rh-endostatin could improve the antitumor effect of adoptive CIK cells transfer and to illustrate the possible mechanisms by which rh-endostatin released the full potential of adoptive cell therapy. Our findings showed that rh-endostatin could improve the antitumor effects of CIK cells and suggested that combination therapy with rh-endostatin and CIK cells held great promise in the treatment of advanced stage lung cancer patients.

## Methods

### Ethics Statement

Animal study was carried out in strict accordance with guidelines for the Care and Use of Laboratory Animals of the Jinling Hospital. The protocol was approved by the Animal Ethics Committee of Jinling Hospital (NO. 2010062412). All surgery was performed under pentobarbital sodium and ketamine anesthesia and animals were sacrificed by overdose of anesthetics. All efforts were made to minimize suffering. Human blood samples were collected from informed donors and the procedure was carried out in strict accordance with the procedure approved by Ethic Committee of Jinling Hospital. Blood sample donors have provided their written informed consents to participate in this study.

### Cell Lines

Murine Lewis lung carcinoma cells, human lung adenocarcinoma cells A549 and SPC-A1 were purchased from Shanghai institute of biochemistry and cell biology (Shanghai, China). HUVECs were purchased from KeyGen Biotech (Nanjing, China). Murine Lewis lung carcinoma cells [19] were maintained in Dulbecco’s Modified Eagle Medium (Invitrogen, USA) complemented by 100 U/ml Penicillin, 100 µg/ml Streptomycin and 10% fetal bovine serum. Human lung adenocarcinoma cells A549 [8] and SPC-A1 [21] were maintained in our laboratory and grown in RPMI-1640 (Gibaco, USA) supplemented with 100 U/ml Penicillin, 100 µg/ml Streptomycin and 10% fetal calf serum. HUVECs [22] were grown in F12K (Mediatech) supplemented with 100 U/ml Penicillin, 100 µg/ml Streptomycin, 10% FBS, 0.1 mg/ml Heparin, 0.03 mg/ml endothelial cell growth supplement (Sigma Aldrich). All cells were cultured at 37°C in a humidified atmosphere of 5% CO_2_.

### Animal Models

SPF C57BL/6 mice and BALB/C nude mice (6–8 weeks old) were purchased from Academy of Military Medical Science (Beijing, China), kept in the Comparative Medicine Department of Jinling Hospital and bred under controlled temperature and humidity, and a 12-hours dark, 12-hours light cycle with sterile food and water ad libitum. We established three animal models in the present study. Two human lung adenocarcinoma xenograft models were established by subcutaneous inoculation of A549 cells and SPC-A1 cells (1×10^6^/µl PBS) respectively into the right flank of BALB/C nude mice. For the third tumor model, C57BL/6 mice were challenged subcutaneously in the right flank with 1×10^6^ Lewis lung carcinoma cells in 100 µl PBS. Subcutaneous tumor volumes were measured daily by caliper and tumor volumes were calculated by the formula: tumor volume = 0.52×length×width^2^.

### Generation and Cellular Phenotype Analysis of CIK Cells

The human CIK cells were generated as previously described [23]. Briefly, blood samples from consenting donors were processed using Ficoll-Hypaque density gradient centrifugation (Beijing Chemical Reagents Company, China) to separate peripheral blood mononuclear cells. After washing in the medium, cells were resuspended in RPMI-1640 medium (10% FCS, 100 U/ml penicillin, and 100 µg/ml streptomycin) at a density of 2–4×10^6^ cells/ml. The 1000 U/ml interferon-γ (Peprotech, USA) was added on the first day of culture. After 24 hours, 500 U/ml Interleukin-2 (Peprotech, USA) and 50 ng/ml anti-CD3 antibody (eBioscience, USA) were added. Cells were maintained at 37°C in a humidified atmosphere of 5% CO_2_ and were sub-cultured every 2–3 days with fresh complete medium with 300 U/ml Interleukin-2 (Peprotech, USA) for 2 weeks. The generation of mouse CIK cells from spleen cells was operated similar to that of human CIK cells. In order to characterize the phenotypes of CIK cells, cells cultured for 7, 14 and 21 days were harvested and stained for 30 min at 4°C with the following FITC or PE-conjugated monoclonal antibodies (mAbs): anti-CD3 and anti-CD56. By flow cytometry, the expression of surface markers, CD3, CD56 were examined and recorded (Fig. S2). All antibodies were obtained from (eBioscience, San Diego, CA) by using standard procedures. A total of 100000 cells per sample were acquired and analyzed on a FACS-Calibur and CellQuest software (BD Biosciences, San Jose, CA, USA).

### Treatment Protocol

BALB/C nude mice were challenged subcutaneously in the right flank with 100 µl (1×10^7^/ml) A549 cells. When tumor volumes were approximately 100 mm^3^, animals were randomly divided into four groups (n = 4–5 per each group), and treatments were initiated. The day when treatment started was designated d0. The antiangiogenesis therapy in this study was subcutaneous injection of 5 mg/kg rh-endostatin for 7 days and the adoptive immunotherapy consisted of two intravenous transfusion of CIK cells at d6 and d9 (2×10^7^ cells per dose in a total volume of 100 µl). The detailed groupings were as follows (Fig. S1): (1) Group NS, treated with normal saline (NS). (2) Group EN, treated with rh-endostatin alone. (3) Group CIK, treated with CIK cells alone. (4) Group EN+CIK, treated with rh-endostatin followed by transfusion of CIK cells. The body weight and tumor volume were recorded daily. The synergism of two therapies was assessed according to the following formula: q = E_A+B_/[E_A_+(1−E_A_) E_B_] [24].

E_A+B_, the inhibition rate of combination therapy.

E_A_, the inhibition rate of rh-endostatin antiangiogenic therapy.

E_B_, the inhibition rate of adoptive CIK cells immunotherapy.

q<0.85 means the two therapies are antagonism.

q>1.15 means the two therapies are synergism.

0.85≤ q ≤1.15 means the two therapies are additive.

The experiment was repeated with four groups of C57B/6 mice carrying Lewis lung carcinoma and four groups of BALB/C nude mice bearing SPC-A1 lung carcinoma.

### Culture Conditions

Hypoxic incubation condition was performed in a humidified, anaerobic work station incubator (Bug Box; ALC International, Cologno Monzese, Milan, Italy). A gas mixture of 1% O_2_, 5% CO_2_, and 94% N_2_ was continuously injected at a flow rate of 25 L/min into the anaerobic work station incubator. For the normoxic condition, cells were cultured at 37°C in a humidified incubator consisting of 20% O_2_, 5% CO_2_, and 75% N_2_. All reagents including medium and plastic materials used for the hypoxic treatments were equilibrated in the anaerobic chamber to minimize contamination with air.

### Carboxyfluorescein Diacetate Succinimidyl Ester (CFSE) Labeling

Labeling of CIK cells with CFSE was performed as reported previously [25]. Briefly, human CIK cells were labeled with CFSE (Molecular Probes Biotec., final concentration of 5 µmol/L in PBS) and incubated in 37°C for 6 minutes. The labeling reaction was quenched by addition of cold RPMI-1640 with 10% FCS, and cells were washed twice with PBS with 2% FCS to remove excess CFSE.

### Suppression of Proliferation Assay

CIK cells were cultured in RPMI-1640 medium (10% FCS, 100 U/ml penicillin, and 100 µg/ml streptomycin) in 96-well plates under normoxia or hypoxia. Anti-CD3 antibody and Interleukin-2 were added into the media in order to stimulate the division of CIK cells. Forty eight hours later, CIK cells were counted by hemocytometry under light microscope.

### In vitro Cytotoxicity Assay

The cytotoxicities of CIK cells against target cells in normoxic and hypoxic conditions were analyzed by using CytoTox 96® Non-Radioactive Cytotoxicity Assay-Lactate Dehydrogenase release assay (Promega, Madison, WI, USA). Briefly, A549 cells were placed in two 96-well plates at 1×10^5^ cells/well and incubated for 48 h with CIK cells at 20∶1, 40∶1 effector-to-target (E-to-T) ratios. A volume of 30 µl cell culture supernatant was transferred to transparent flat-bottom 96-well plates followed by adding 30 µl substrate. After incubating for 30 min in the dark at room temperature, the reaction was stopped by adding 30 µl stop solution. Thereafter, absorbance was measured at 490 nm. The maximal release of LDH was performed by completely lysing target cells. Target cells or effector cells alone were used as negative controls (spontaneous release). The killing rate was determined according to the formula: killing rate (%) = [(experimental counts – effector control counts –target spontaneous counts)/(target maximal counts – target spontaneous counts)]×100.

### Transmigration Assay

Transmigration assay was performed with Transwell™ (Costa, USA) inserts containing HUVECs culture. HUVECs were added to the upper chamber and incubated under normoxic or hypoxic condition for 24 h, respectively. A total of 1×10^6^ CIK cells in 200 µl RPMI-1640 medium were then added upon the HUVECs layer with A549 cells culture supernatant added in the lower chamber and were then incubated under normoxic or hypoxic conditions for 48 h, respectively. CIK cells that migrated into the lower chamber were harvested and counted by hemocytometry.

### Immunocytochemistry

After incubation in hypoxic or normoxic culture condition for 48 h, HUVECs were fixed with 70% ethanol for 10 min and permeabilized by 0.1% TritonX-100 for 5 min. 5% BSA in PBS was used to block the non-specific binding of antibodies. Anti-ICAM-1 antibody (Abcam, Cambridge, MA) and anti-VCAM-1 antibody (Abcam, Cambridge, MA) were used to detect the expression of intercellular cell adhesion molecule-1 (ICAM-1) and vascular endothelial cell adhesion molecule-1 (VCAM-1) by HUVECs. Images were acquired by using Olympus BX-60 microscope at 400× magnification.

### CIK Cells Adhesion to Endothelial Cells in vitro

Subconfluent monolayers of HUVECs in 6-well plates were preincubated in hypoxic or normoxic culture condition for 48 h. Following preincubation, CIK cells (2×10^6^ cells/well) were transferred to the HUVECs cultures and incubated for 24 h under the same culture condition as the HUVECs. Detached cells were removed by two careful washes using PBS and the remaining cells were stained by rabbit anti-mouse CD3 antibodies (Abcam, Cambridge, MA) to detect CIK cells. Images of adherent cells were acquired by using Olympus BX-60 microscope at 200× magnification.

### Tumor Hypoxia Measurement

The hypoxyprobe™-1 kit (Natural Pharmacia International, Inc, Burlington, MA, USA, constituted of 100 mg solid pimonidazole HCl (hypoxyprobe™-1) and 1.0 ml mouse IgG1 monoclonal antibody (Mab1)) were used to further study the effect of rh-endostatin on tumor hypoxia. The basis of this measurement is that pimonidazole is reductively activated when the tissue pO_2_ is below 10 mmHg and the activated intermediate forms stable covalent adducts with thiol groups in proteins, peptides and amino acids. These adducts can be detected by immunochemical means when they are combined with the antibody reagent Mab1. For tumor hypoxia detection, mice burdened with A549 lung carcinoma were given 60 mg/kg body weight pimonidazole HCl (hypoxyprobe™-1) intraperitoneally 4 h before being sacrificed at three different time points (days 3, 6, 9). Tumors were dissected and fixed in 10% formalin, embedded in paraffin and then stained with a mouse IgG_1_ antibody. Images of the sections were acquired by using Olympus BX-60 microscope at 100× magnification and hypoxic areas were analyzed using digital image analysis software Image J (NIH, Maryland, USA).

### In vivo Tracking of CIK Cells

Seven days after the administration of rh-endostatin, A549 tumor-bearing mice were transfused i.v. with CFSE-labeled CIK cells (2×10^7^ cells in a total volume of 100 ul). Normal saline was used as negative control (n = 4 animals per group). Twenty four hours after CIK cells transfusion, mice were sacrificed and single cell suspensions of spleen and tumor tissue were prepared from two groups. Analysis of the percentage of CFSE-labeled CIK cells was performed on a FACS-Calibur (BD Biosciences, San Jose, CA, USA). Ten thousands gated events were collected and analyzed using CellQuest software (BD Biosciences, San Jose, CA, USA).

### Flow Cytometry

Seven days after the treatment of rh-endostatin, tumor-bearing C57BL/6 mice were sacrificed. Tumors and spleens were harvested and single cell suspensions were prepared. Erythrocytes were removed after incubation in erylysis buffer [155 mmol/L NH4Cl, 10 mmol/L KHCO3, and 0.1 mmol/L EDTA (PH 7.4)] at room temperature for 10 min. Cells were stained for 30 min at 4°C with the following FITC or PE-conjugated monoclonal antibodies (mAbs): anti-CD11b and Gr-1 for Myeloid-derived suppressor cells (MDSCs), anti-F4/80 and MHC/II for tumor-associated macrophages (TAMs). All antibodies were obtained from (eBioscience, San Diego, CA) by using standard procedures. A total of 100000 cells per sample were acquired and analyzed on a FACS-Calibur and CellQuest software (BD Biosciences, San Jose, CA, USA).

### Intravital Microscopy and Vascular Permeability Assay

A549 tumor-bearing mice were treated with rh-endostatin (5 mg/kg, s.c.) for consecutive 7 days with normal saline as control. On days 3, 6 and 9, mice were anesthetized by intraperitoneal administration of pentobarbital sodium (40 mg/kg body weight) and ketamine (20 mg/kg body weight). Briefly, mice were kept warm using heating pads throughout the surgical procedure. One layer of the skin (12 mm) around the tumor was surgically removed to create an observation window. After addition of sterile PBS, a thin sterile contact lens was used to cover the surgical site and provide visual access to the vascular network of the tumor (Fig. S3A). After the surgery, the mouse was fixed under fluorescence microscope and the interested vasculature was observed. After the i.v. injection of 20 mg/kg Evans Blue dye (Sigma-Aldrich, USA), the distribution course of Evans blue was recorded for about 10 minutes. The extravasation of Evans Blue was analyzed in the recorded video. The data were confirmed by Evans Blue extraction test after the termination of intravital microscopy examination.

### Immunohistochemistry

C57BL/6 mice were challenged subcutaneously in the right flank with Lewis lung carcinoma cells. When tumor volumes were approximately 100 mm^3^, animals were randomly divided into four groups according to the treatment protocol, and treatments were initiated on day 0. On day 14, tumors from all experiment groups were removed and tumor samples were fixed in 4% formalin and embedded in paraffin for immunohistochemistry studies. Adjacent 3-µm sections were made and stained with hematoxylin and eosin (H&E). Rabbit anti-mouse CD3 antibodies (Abcam, Cambridge, MA) were used to detect T lymphocytes. Images of sections were acquired by using Olympus BX-60 microscope at 200× and 400× magnification. The number of CD3^+^ T lymphocytes was obtained by counting the CD3-positive stained cells. At least ten random fields were evaluated for each section by two observers independently. Consensus results were used for the final numbers of T lymphocytes.

### Immunofluorescence

A549 tumor samples were fixed in 4% formalin, embedded in paraffin and adjacent 3-µm sections were made for immunofluorescence studies. Slides were deparaffined in xylene and rehydrated in graded ethanols and rinsed in dH_2_O. Slides were boiled for 2 minutes in citrate buffer (Zymed), cooled for 15 minutes and then washed in PBS for 5 minutes. Specimens were incubated for 1 hour in a blocking solution, and then incubated with primary antibodies at 4°C overnight. Rat anti-mouse CD31 antibodies (1∶50; Abcam, Cambridge, MA) were used to stain vascular endothelial cells, rabbit anti-mouse alpha smooth muscle actin (α-SMA) antibodies (1∶200; Abcam, Cambridge, MA) were used to stain pericytes. Tumor tissues were double-stained with anti-CD31 antibodies and anti-α-SMA antibodies. Slides were then rinsed in PBS and incubated in secondary antibodies for 2 h at room temperature in the dark. Secondary antibodies were goat anti-rat FITC-conjugated antibodies and goat anti-rabbit TRITC antibodies. Slides were then rinsed in PBS. Images of the sections were captured. Microvascular density (MVD) was assessed as previously described by two observers independently [26]. In brief, the sections were screened at lower magnifications (100×) to identify the ten areas containing the highest number of capillaries and small venules. Within the selected area, CD31-positive stained endothelial cells (MVD) and CD31/α-SMA double-stained vessels were counted at a magnification of ×400.

### Dynamic Contrast Enhanced Magnetic Resonance Imaging (DCE-MRI)

A549 tumor-bearing mice were treated with rh-endostatin (5 mg/kg, s.c.) for consecutive 7 days with normal saline as control. On days 3, 6 and 9, mice were anesthetized and DCE-MRI was conducted. DCE-MRI was performed using a 3 tesla whole-body MR-scanner (Siemens Symphony) in combination with a small animal coil for excitation and signal reception. Morphologic MR-imaging was performed using a transversal T2-weighted turbo-spin echo sequence (repetition time, TR 650 ms, echo time, TE 24 ms, field of view, FOV 70×70 mm2, slice thickness 2 mm). T1 map of the tumor section was obtained with 3 Flip angles (5 degree, 15 degree and 30 degree), and T1 values were calculated by curve estimation. Kinetics of the contrast agent in tumors were recorded using a T1-weighted inversion-recovery FLASH sequence (TR 5 ms, TE 2 ms, slice thickness 5 mm, FOV 55×70 mm2). After starting the DCE-MRI measurement, 0.1 ml (0.1 mmol/kg) of the paramagnetic contrast agent gadolinium diethylene-triamine penta-acetic acid (Gd-DTPA) (Bayer-Schering Pharma) was injected manually within 5 s into the tail vein, 70 dynamic scans were acquired from one section. Data were analyzed using the pharmacokinetic model as previously reported and parametric images of K^trans^ (the volume transfer constant of the contrast agent) were produced by pharmacokinetic analysis of the DCE-MRI series [27].

### Statistical Analysis

Data were expressed as mean ± standard error (SE). Differences between four groups were compared by ANOVA, and LSD was applied for multiple means comparisons. The single-measurement comparisons between two groups were tested using unpaired t-tests. Pearson correlation analysis was used to test the correlation between intra-tumoral hypoxia and MDSC accumulation. Values of p<0.05 were taken as significant. Statistical analysis was conducted using the SPSS software 16.0 (Chicago, IL, USA).

## Results

### Morphology Observation and Cellular Phenotype of CIK Cells

Under microscopy, CIK cells showed cluster-like growth. Masses of CIK cells gradually multiplied and became larger after 4 days incubation. There are two main subpopulations of CIK cells, one expressing both CD3 and CD56 molecules (CD3^+^CD56^+^) and the other presenting a CD3^+^CD56^−^ phenotype. After incubation for 7, 14 and 21 days, phenotypes of CIK cells were detected. The percentages of CD3^+^CD56^+^ CIK cells were 8.93%, 23.66% and 17.17% on day 7, 14 and 21, respectively (Fig. S2).

### Combination of rh-endostatin with Adoptive CIK Cells Transfer Significantly Inhibits the Growth of Lung Carcinoma in vivo

**Figure 1:**
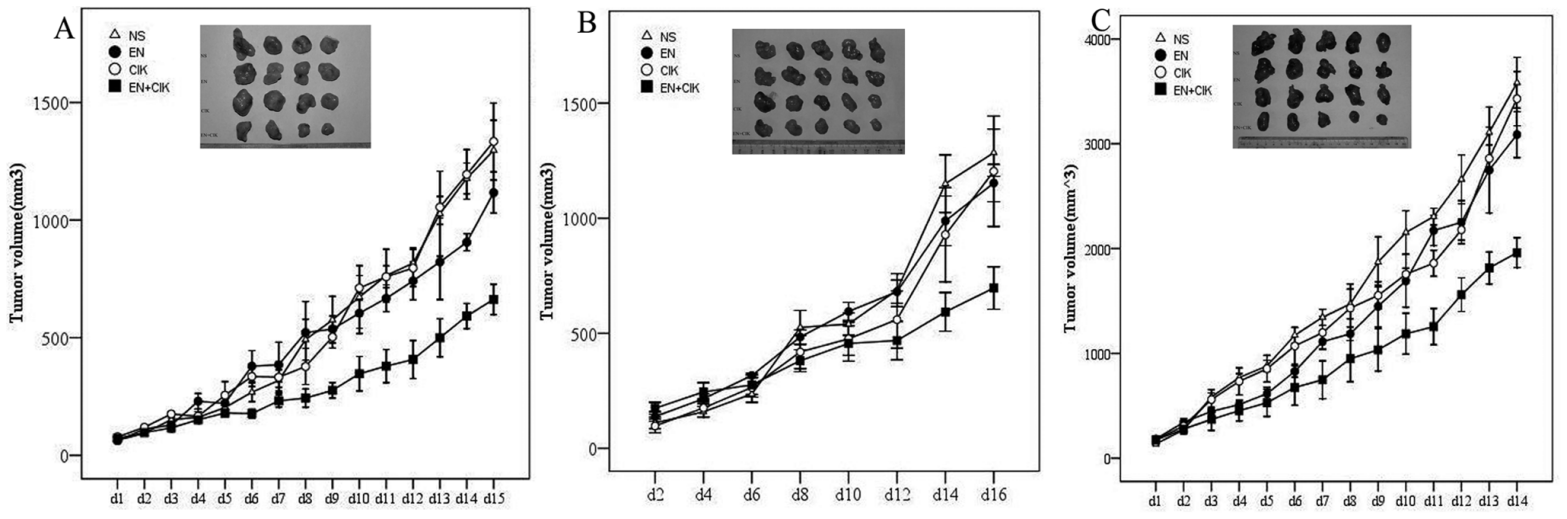
Synergistic anti-tumor effect of rh-endostatin and adoptive CIK cells therapy on tumor growth. BALB/C nude mice were injected s.c. with A549 lung cancer cells and when tumor volumes reached 100 mm^3^, mice were divided into four groups randomly and received respective protocols according to the treatment schema. SPC-A1 and Lewis lung carcinoma xenografts were established and given similar treatments. A, synergistic anti-tumor effect was shown when rh-endostatin was combined with CIK adoptive therapy in suppressing A549 tumor growth (p<0.05). Rh-endostatin or CIK therapy alone did not significantly suppress tumor growth. B, rh-endostatin exhibited a substantial antitumor effect when given in combination with CIK cells in inhibiting SPC-A1 lung carcinoma (p<0.05). C, synergistic anti-tumor effect was shown when rh-endostatin was combined with CIK adoptive therapy in suppressing Lewis lung carcinoma growth (p<0.05). Points, means of tumor volumes of mice per group; Bars, SE. Results shown are representative of three independent experiments.

To determine whether the addition of rh-endostatin has any synergistic effect on the antitumor efficacy of CIK cells, three representative tumor models were established. In A549 lung carcinoma model, we found that rh-endostatin slightly limited tumor growth compared with NS control, but did not reach statistical significance (929.46±471.70 vs 1251.8±531.75, p = 0.324). CIK cells adoptive therapy alone had no antitumor activity compared with NS control (1192.1±621.68 vs. 1251.8±531.75, p = 0.852). On the contrary, significant (p<0.05) antitumor activity was seen after combination therapy compared with the monotherapy or the NS control (Fig. 1A). E_A_ = 1.35, E_B_ = 1.05, E_AB_ = 2.08 and q = 2.71, which confirms the assessment of synergism. Similar results were observed in established Lewis and SPC-A1 lung carcinoma models. In SPC-A1 lung cancer model, compared with NS control, rh-endostatin (1152.8±181.4 vs 1284.7±229.2, p = 0.527) and CIK (1204.1±536.2 vs 1284.7±229.2, p = 0.698) monotherapy did not show any significant antitumor effect. However, significant antitumor effect was achieved in combination therapy (p<0.05) (Fig. 1B). In Lewis lung carcinoma, neither rh-endostatin (3394.7±668.4 vs 3866.5±490.62, p = 0.176) nor CIK cells therapy (3429.6±579.12 vs 3866.5±490.62, p = 0.208) alone exhibited obvious inhibition on tumor growth compared with the NS control. However, significant synergistic antitumor effect was shown when rh-endostatin was added to CIK therapy (2037.0±294.6 vs 3866.5±490.62, p = 0.000) (Fig. 1C). In vivo experiments were repeated twice. And similar results were got in three different lung carcinoma models all of which suggested that synergetic antitumor effects were obtained by combining of rh-endostatin antiangiogenic therapy and CIK cells adoptive therapy.

### Rh-endostatin Decreases Microvascular Density and Promotes Tumor Vessel Normalization

**Figure 2:**
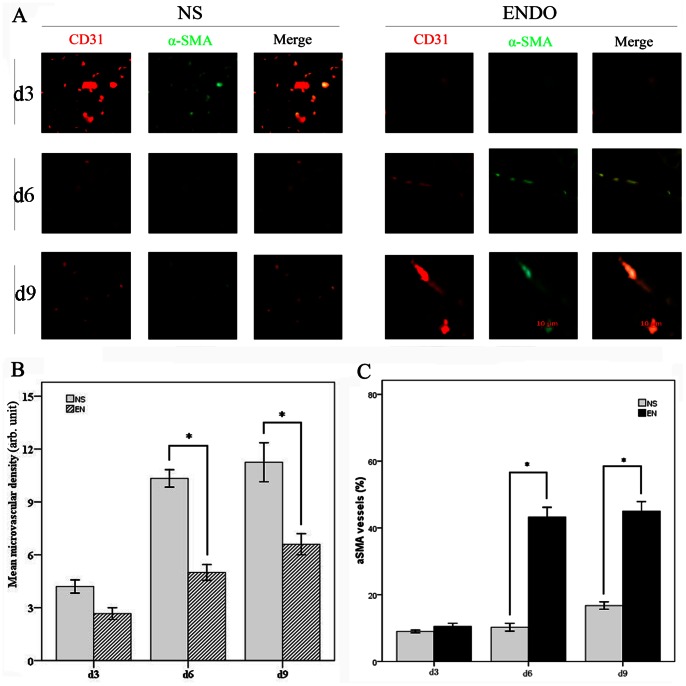
Rh-endostatin decreases microvascular density (MVD) and promotes vessel normalization of A549 lung carcinoma. A549 tumor-bearing mice were treated with rh-endostatin (5 mg/kg, s.c.) for consecutive 7 days with normal saline as control. On days 3, 6 and 9, mice (n = 4, each group and each time point) were sacrificed and tumor samples were stained by anti-CD31 antibody and anti-α-SMA antibody. A, typical fluorescence images of tumors showed CD31-positive endothelial cells (red), α-SMA positive pericytes (green) and merged images (orange) in A549 lung carcinoma in control group and rh-endostatin treated group on days 3, 6, and 9, respectively. B, columns represented microvascular densities at different time points in two groups. C, columns represented pericyte coverages at different time points in the two groups. Representative sections are shown from all groups with a magnification of 400×. Columns, mean; Bars, SE; *p<0.05, indicating significant difference.

In our previous study, we found that microvascular density was decreased and collagen coverage was increased by rh-endostatin in murine Lewis lung carcinoma bearing C57BL/6 mice [19]. In the present study, microvascular density (MVD) was evaluated in A549 tumor bearing mice. On day 3, 6 and 9, mice from each group were sacrificed and tumor samples were prepared for immunofluorescence staining. Staining for endothelial cell marker CD31 showed that, the MVD was reduced on day 6 and 9 in the rh-endostatin treated group compared with those in NS control (p = 0.042 and 0.036, respectively, Fig. 2A and B). Since increased pericyte coverage was considered as a key characteristic of tumor vessel normalization, we double stained CD31 and the pericyte marker α–smooth muscle actin (α-SMA). Notable increase in pericyte coverage of tumor vessels was found in rh-endostatin treated group on day 6 and 9 (p = 0.027 and 0.035, respectively), while no significant difference was observed on day 3 (p = 0.257, Fig. 2A and C). Overall, rh-endostatin induces structural normalization of tumor vasculature by decreasing tumor angiogenesis and improving tumor vessel maturation.

### Rh-endostatin Treated Tumors Show Increased K^trans^ Value for Gd-DTPA and Delayed Evans Blue Extravasation

**Figure 3:**
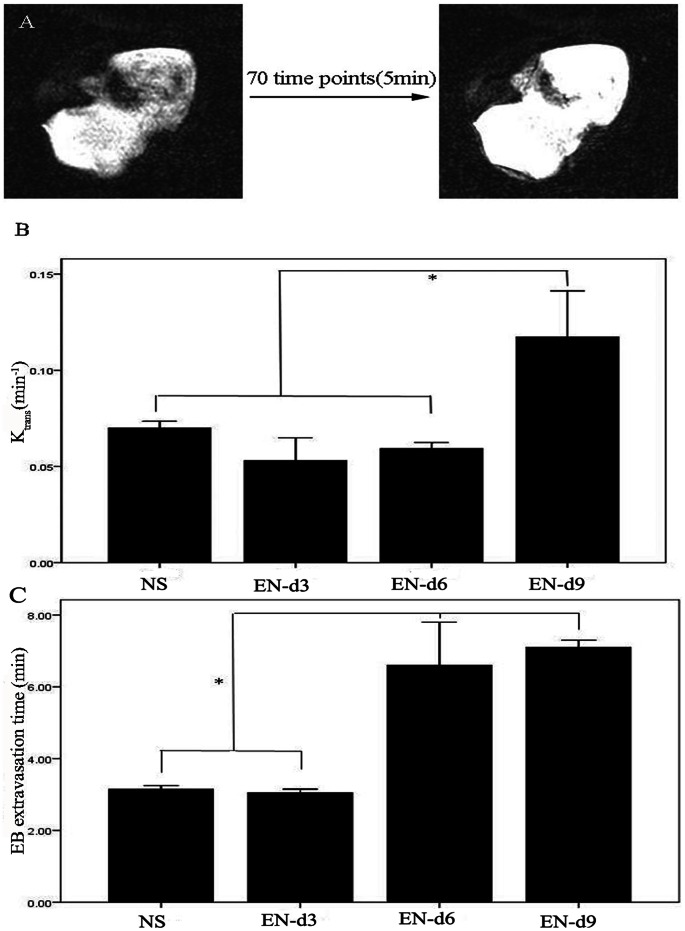
Rh-endostatin treated tumors show increased K^trans^ value for Gd-DTPA and delayed Evans blue extravasation. DCE-MRI and intravital microscopy were performed to test tumor vascular permeability to small molecules and macromolecules, respectively. A, representative images of DCE-MRI with Gd-DTPA well infiltrating in the A549 tumor. B, the columns represent mean ± SE of K^trans^ for A549 tumors in control group and rh-endostatin treated group at day3, 6 and 9. C, the columns represent mean ± SE of Evans blue extravasation time in control group and rh-endostatin treated group at day 3, 6 and 9. Columns, mean; Bars, SE; *p<0.05 indicating statistical significance. Results shown are representative of 3 independent experiments.

**Figure 4:**
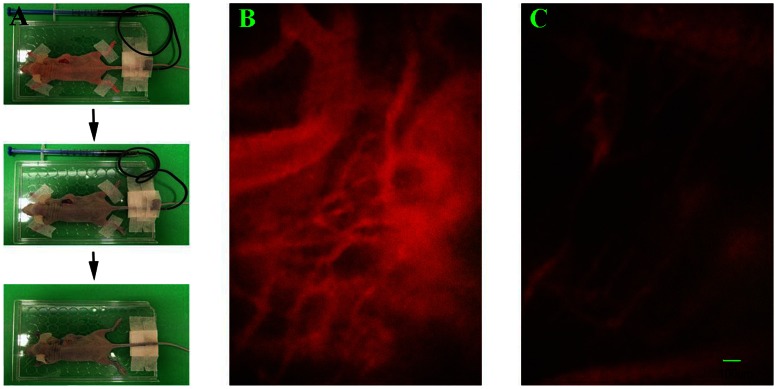
Tumor vascular is normalized by rh-endostatin assessed by intravital microscopy. A549 tumor-bearing mice were treated with rh-endostatin (5 mg/kg, s.c.) for consecutive 7 days with normal saline as control. On days 3, 6 and 9, intravital microscopy were performed to test tumor vascular permeability to macromolecules. A, representative figures showing the exposure of tumor surface and intravenous injection of Evans blue in to BALB/c mice. B, a representative figure of Evans blue infused tumor vessels of control group at 100× magnification. C, a representative figure of Evans blue infused tumor vessels of rh-endostatin treated group at 100× magnification.

To clarify whether structural normalization of tumor vessels translates to functional normalization, we measured tumor vessel permeability, a functional parameter governing the transport of molecules across the vessel wall. A549 tumor bearing mice were injected with Gd-DTPA i.v. and conducted the DCE-MRI. Gd-DTPA (molecular weight 0.55 kD) was well infiltrated in the tumor, as indicated in Fig. 3A. The parameter K^trans^ for Gd-DTPA, representing vascular permeability to small molecules, was significantly increased by rh-endostatin at day 9 (p<0.05, Fig. 3B), prognosticating a higher penetration for drugs and oxygen into the tumor microenvironment. We then examined the tumor vascular permeability to macromolecules by intravital microscopy which allowed us to observe the diffusion of Evans blue-Albumin (molecular weight about 67 kD) from tumor vessel into tumor parenchyma. After the injection of Evans blue, the vasculature was immediately perfused with Evans blue and turned red (Fig. S3C). Evans blue was combined with albumin and formed Evans blue-Albumin as soon as it was injected into tumor vessels (Fig. S3C). Evens blue-Albumin could extravasate leaky tumor vessels and the time when clearly shown vessels became blurry was defined as the point when Evans blue-Albumin extravasated from tumor vessel into tumor parenchyma (Fig. S3D and E). The longer time that it takes Evens blue-Albumin to extravasate from tumor vessels into tumor parenchyma indicates the less abnormal tumor vascular hyperpermeability. The extravasation of Evans blue-Albumin was significantly delayed by rh-endostatin, especially after administration of rh-endostatin for 6 days (p<0.05, Fig. 3C), prognosticating a decreased interstitial fluid pressure. Tumor vessels were less tortuous and less dilated after the administration of rh-endostatin (Fig. 4C) compared with NS controls (Fig. 4B). The decreased tumor vessel permeability to macromolecules and increased permeability to small molecules are hallmarks of tumor vascular normalization.

### Rh-endostatin Decreases Hypoxic Area in Tumor Microenvironment and in vitro Hypoxic Culture Condition Inhibits the Proliferation, Cytotoxicity and Migration of CIK Cells

**Figure 5:**
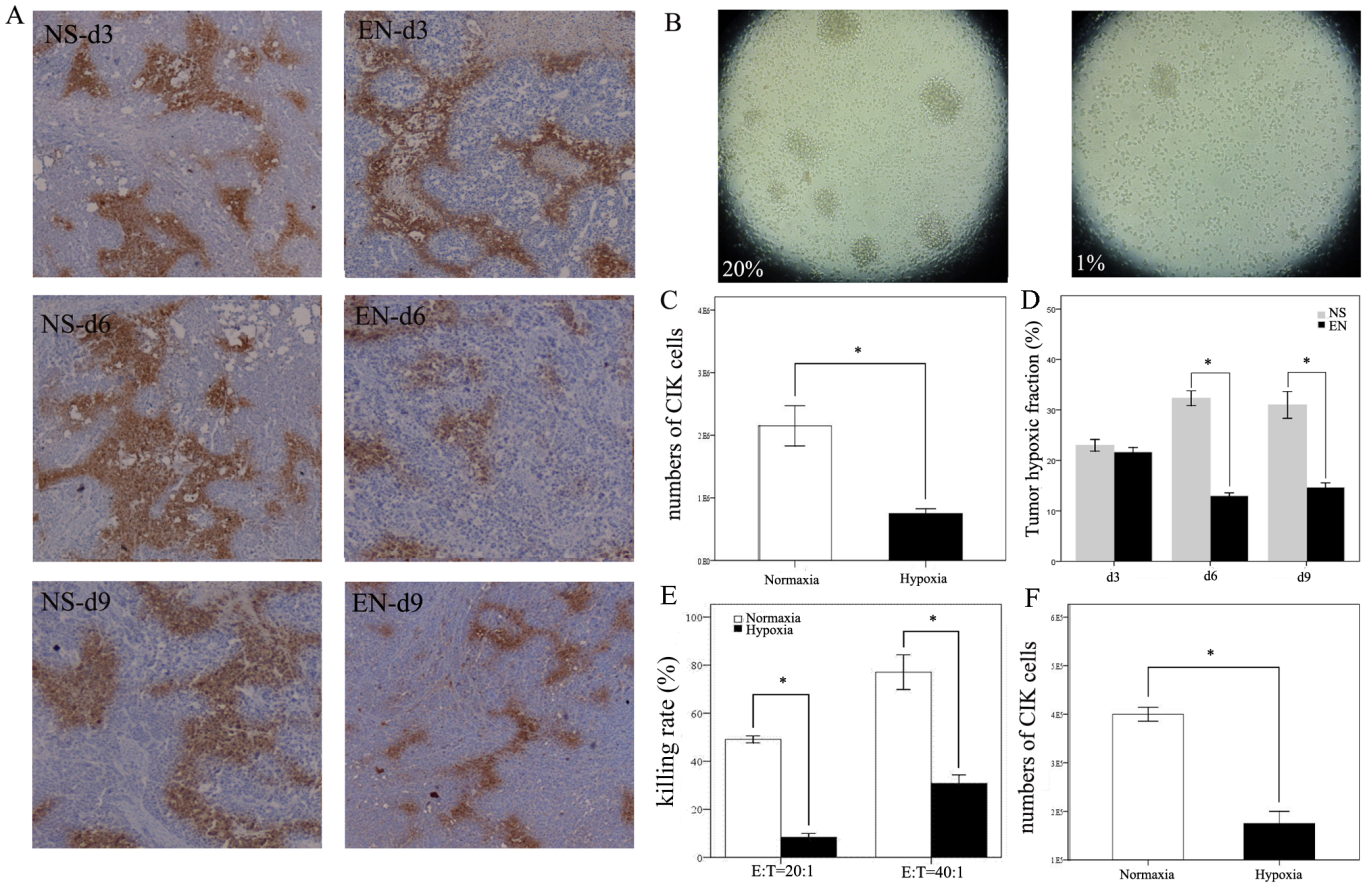
Rh-endostatin decreases tumor hypoxic area and hypoxia inhibits the activity of CIK cells in vitro. A549-bearing mice were divided into two groups and were given rh-endostatin or normal saline respectively for 7 days. On days 3, 6 and 9, mice (n = 4) in the two groups were administrated with pimonidazole. Tumor hypoxic areas were stained by monoclonal antibody (Mab1) against protein adducts of pimonidazole. Hypoxic areas were stained dark yellow. In vitro experiments were conducted by culturing CIK cells under normoxia or hypoxia for 48 h. Proliferation of CIK cells were measured by counting cells with a hemocytometer. A549 lung cancer cells were cocultured with the indicated number of CIK cells under normoxia or hypoxia for 48 h in 96-well plates. The killing rate of CIK cells against the A549 lung cancer cell was measured by LDH release assay. Transmigration assay was performed with Transwell™ inserts containing HUVECs culture. CIK cells were added upon the HUVECs layer and were incubated under normoxic or hypoxic conditions for 48 h. CIK cells that migrated into the lower chamber were harvested and counted by hemocytometry. A, Individual fields at 40×magnification were chosen to represent hypoxic areas in tumor samples on days 3, 6 and 9 in two groups. B, representative pictures of CIK cells cultured under normoxia or hypoxia at 100×magnification. C, bar graphs depicting the density of CIK cells cultured under normoxia or hypoxia. D, bar graphs depicting hypoxic fraction in rh-endostatin treated group or control group. E, bar graphs depicting the cytotoxicity of CIK cells towards A549 cells at different E-to-T ratio. F, bar graph depicting the number of CIK cells migrating across the HUVECs layer. Similar results were observed in three independent experiments. Columns, mean; Bars, SE; *p<0.05 indicating statistical significance.

Hypoxia is caused by poor blood perfusion and poor oxygen diffusion. From the data above, we found that rh-endostatin could normalize the structure and function of tumor vasculature. We then tested whether alteration in tumor vessel structure was helpful to reduce hypoxic area in the tumor microenvironment. Although, no significant difference was found in the hypoxic area on day 3, treatment of rh-endostatin resulted in less hypoxic area, as assessed by pimonidazole staining on day 6 and day 9 in A549 lung carcinoma compared with NS controls (p = 0.002 and p = 0.0093, respectively, Fig. 5A and D). We also analyzed spacial relationship between tumor hypoxic area and the density of tumor infiltrating CIK cells. Continuous section slides were made and stained for hypoxia and CD3^+^ CIK cells, respectively (Fig. S4A and B). More CD3^+^ CIK cells accumulated in normoxic tumor tissue (Fig. S4C) while fewer CIK cells could be found in hypoxic tumor tissue (Fig. S4D and E). As the essential steps in the adoptive immune response, T cell activation and proliferation are critical events needed for antitumor immune surveillance. In the next set of experiments, we cultured CIK cells in 20% or 1% oxygen for 48 h to evaluate the capacity of CIK cells to proliferate under normoxia and hypoxia. Proliferation of CIK cells was determined by counting cells with a hemocytometer, using trypan blue to distinguish live and dead cells. Under light microscopy, CIK cells formed larger and more cell spheres (Fig. 5B) and had a higher rate of proliferation under normoxia compared with hypoxia (p<0.05, Fig. 5C). We next tested whether the alternation in oxygen level would affect the cytotoxicity of CIK cells against A549 lung cancer cells. After coculturing CIK cells and A549 lung cancer cells at 20:1 and 40∶1 E-to-T ratios under normoxia or hypoxia for 48 hours, the killing rates were analyzed by Lactate Dehydrogenase release assay. Under normoxia, CIK cells showed moderate cytotoxicity on A549 cells, with 49.09±2.54% killing rate at 20∶1 E-to-T ratio. However, hypoxia significantly decreased the cytotoxicity of CIK cells with 8.35±2.74% killing rate at 20∶1 E-to-T ratio (Fig. 5E). Similarly, hypoxia notably inhibited the cytotoxicity of CIK cells against A549 cells at 40∶1 E-to-T ratio (30.76±6.19% vs. 77.06±12.48%, p = 0.005, Fig. 5E). Transmigration assay showed that the number of CIK cells migrating HUVECs layer under normoxia was 2.89 times more than that under hypoxia (p = 0.003, Fig. 5F). Except for inhibiting the proliferation and cytotoxicity of CIK cells, hypoxia impeded the migration of CIK cells across the HUVECs layer. In vivo study showed that hypoxia could impede the recruitment of CIK cells into tumor. We next explored the possible mechanisms by culturing HUVECs under hypoxic or normoxic culture conditions. We found that hypoxia could impede the adhesion of CIK cells to HUVECs (Fig. S5). In addition, hypoxia depressed the expression of ICAM-1 and VCAM-1 on HUVECs (Fig. S5). We further explored the spacial relationship between CIK cells infiltration and the endothelial cell adhesion molecules within the tumor ex vivo. Continuous section slides were made and stained for ICAM-1, VCAM-1 and tumor infiltrating CIK cells respectively. We found that the accumulation of tumor infiltrating CIK cells was correlated with the expression of ICAM-1 and VCAM-1. More CIK cells accumulated around the ICAM-1 high expression vessels. Similarly, more CIK cells were found around VCAM-1 high expression vessels compared with VCAM-1 low expression vessels (data are not shown). Our results could possibly explain the decreased recruitment of CIK cells into hypoxic tumor tissue in vivo and reduced CIK cells adhesion to HUVECs in vitro.

### Rh-endostatin Augments the Homing of CIK Cells and the Intratumoral CD3^+^ T lymphocytes are Increased after the Combination Therapy

**Figure 6:**
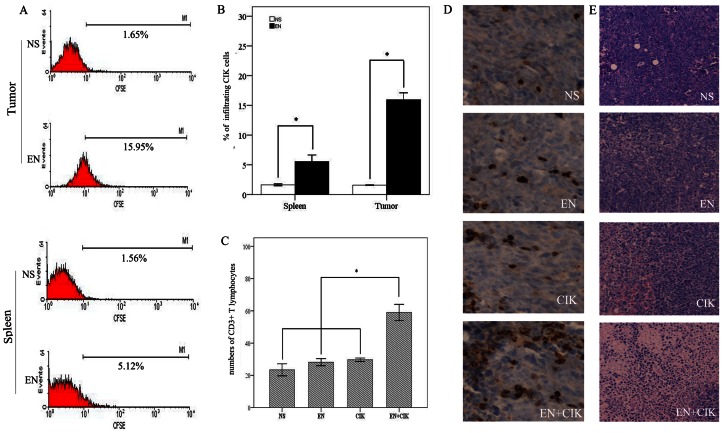
Lymphocytes accumulation in the tumor or spleen of tumor bearing mice. A, A549 tumor-bearing mice were treated with normal saline or rh-endostatin (5 mg/kg, s.c.) for 7 days. CFSE-labeled 2×10^7^ CIK cells were transfused i.v. into 4 mice from each group on d6 (n = 4). Twenty four hours after CIK cells transfusion, mice were sacrificed and single cell suspensions of spleen and tumor tissue were prepared. CIK cells infiltration was analyzed by flow cytometry. Flow cytometry analysis data showing the percentages of CFSE-labeled CIK cells infiltrating into the tumor and spleen after the administration of normal saline or rh-endostatin. B, the columns represent mean ± SE of CFSE-labeled CIK cells infiltrating percentages into the tumor and spleen of the hosting mice in two groups. C, C57BL/6 mice were injected s.c. with Lewis lung carcinoma cells and the treatment protocols were initiated when tumor volume reached 100 mm^3^. On day 14, mice were sacrificed and tumor sections were prepared and analyzed by CD3 staining. Ten individual fields surrounding the apoptotic area at 400× magnification were chosen to enumerate the numbers of intratumoral CD3^+^ T lymphocytes. D, representative sections are shown from all groups at 400× magnification. E, histological H&E staining showing tumor necrosis from each group at 200× magnification. Columns, mean; Bars, SE; *p<0.05 indicating statistical significance.

Since hypoxia inhibited the migration and adhesion of CIK cells in vitro, we then tested whether the homing of CIK cells was impeded in vivo under hypoxic tumor microenvironment and whether the synergistic antitumor effect of the combination therapy was associated with an enhanced CIK cells infiltration into the tumor and spleen of tumor-bearing mice. We have established two kinds of tumor models in our present research. One was in immunodeficient mice and the other was in immunocompetent mice. A549 lung cancer-bearing mice were infused with CFSE-labeled CIK cells after rh-endostatin treatment for 7 consecutive days with normal saline as control on day 6. There was 15.95±1.64% CFSE-labeled CIK cells infiltrating into tumor from treatment group compared with 1.57±0.07% from control group (p = 0.006, Fig. 6A and B). CIK cells infiltration into the spleen was also increased in rh-endostatin treated group (5.55±1.56% vs 1.63±0.22%, p = 0.036, Fig. 6A and B). In the following experiment, the accumulation of CD3^+^T lymphocyte was examined in Lewis lung carcinoma xenograft. On day 14, tumors from all experiment groups were removed and CD3 was used as a specific marker to enumerate T lymphocytes. Only a few lymphocytes accumulated in the tumor of control group. Rh-endostatin and CIK cells therapy alone slightly increased the level of infiltrating CD3^+^ T lymphocytes but did not reach statistical significance. However, tumor-infiltrating CD3^+^ T lymphocytes were significantly improved in the combinatorial group (Fig. 6C and D) which was consistent with the delayed tumor growth. In order to analyze CIK cells antitumoral activity in vivo, tumor necrosis was tested by H&E stain. H&E stain section from control tumor showed no regions of necrosis. Sections from rh-endostatin or CIK cells treated tumors showed minor degree of necrosis. However, tumor from combinational group showed large area of liquefactive necrosis surrounded by viable tumor (Fig. 6E).

### Rh-endostatin Inhibits the Accumulation of MDSCs in the Tumor without Influencing the Infiltration of TAMs

**Figure 7:**
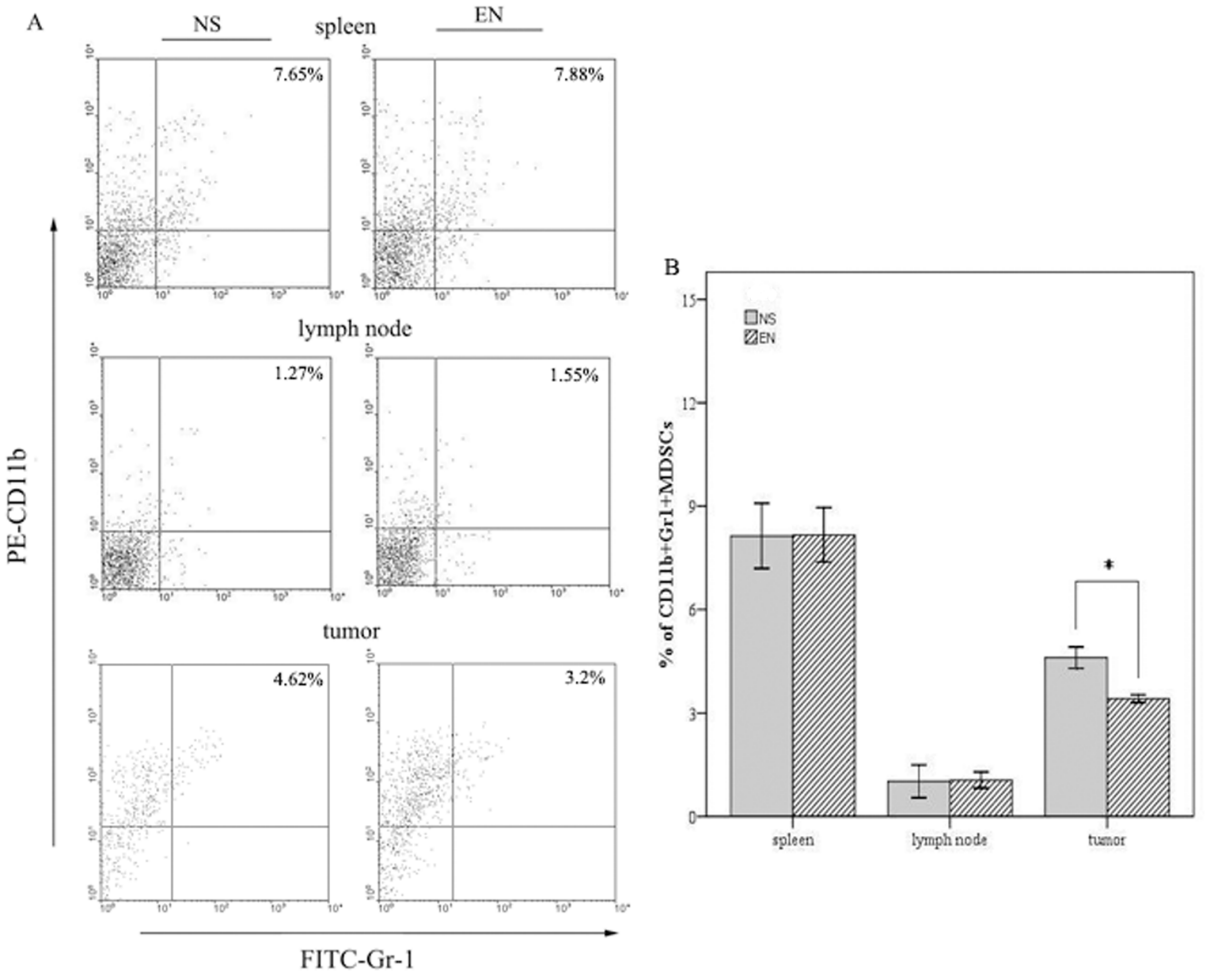
Rh-endostatin depletes the accumulation of MDSCs in the tumor. C57BL/6 mice were injected s.c. with Lewis lung carcinoma cells and when tumor volume reached 100 mm^3^ treatment were initiated. After administration of rh-endostatin for consecutive 7 days, tumor-bearing mice were sacrificed and single cell suspensions of spleen, lymph node and tumor tissue were prepared to analyze frequency of MDSCs by flow cytometry. A, representative flow cytometry analysis data showing the frequency of CD11b^+^Gr1^+^ MDSCs in two groups. B, bar graph depicting the percentages of MDSCs in the spleen, lymph node or the tumor. Columns, mean; Bars, SE; *p<0.05 indicating statistical significance.

We next explored whether rh-endostatin affect the immune response. We focused on MDSCs and TAMs as their accumulation were associated with tumor growth. After 7 days of consecutive administration of rh-endostatin or normal saline, Lewis lung cancer bearing mice were sacrificed and single cell suspensions of spleen, lymph node and tumor tissue were analyzed by flow cytometry. We observed that rh-endostatin was able to significantly decrease the CD11b^+^Gr-1^+^ MDSCs frequency in the tumor (3.21±0.19% vs 4.62±0.43%, p = 0.022, Fig. 7A and B). However, after the treatment of rh-endostatin, MDSCs frequency in the spleen of tumor-bearing mice was comparable with its control (7.65±1.65% vs 7.82±1.51%, p = 0.394, Fig. 7A and B). No obvious difference was obtained in the lymph node (1.75±1.96% vs 1.77±1.47%, p = 0.984, Figs. 7A and B). No difference was shown in the infiltration of TAMs in tumor, spleen and lymph node between rh-endostatin treated group and control group (Fig. S6). Pearson correlation analysis was used to test the correlation between intra-tumor hypoxia and MDSC accumulation. The intra-tumoral MDSC accumulation was directly related with intra-tumoral hypoxia (correlation coefficients = 0.879, p = 0.011).

## Discussion

CIK cells are a heterogeneous subset of ex-vivo expanded T lymphocytes which are characterized with a MHC-unrestricted tumor-killing activity and a mixed T-NK phenotype. Emerging preclinical and clinical studies have suggested that adoptive transfer of CIK cells was a promising immunotherapy for hematologic and solid malignancies including melanoma, colorectal, renal carcinoma, gastric carcinoma and non-small cell lung cancer [2], [3], [28]. However in clinical studies, the therapeutic activity of CIK cells transfer is limited due to escape of tumor cells from immune destruction, lack of costimulatory molecule expression on tumors [29]. In addition, tumors have the capacity to reduce the activity of lymphocytes and limit the migration of lymphocytes from tumor vessels into the tumor parenchyma because of its immunosuppressive microenvironment and the morphologically abnormal and functionally defective tumor vasculature [30], [31]. Anti-VEGF antibody has been reported to increase lymphocyte infiltration into tumor and to enhance the effectiveness of adoptive immunotherapy of cancer [16]. Delivery of adenoviruses encoding antiangiogenic factors (pigment epithelium-derived factor and endostatin) synergized with cytokines (GM-CSF and IL-12) to induce an significant therapeutic effect against hepatocellular carcinoma by reducing immune tolerance and making tumor more vulnerable to the immune reactions [15].

Our previous work showed that rh-endostatin normalized tumor vasculature and microenvironment and significantly reduced non-necrotic hypoxic fraction in Lewis lung carcinoma [19]. In the present study, we initiated the combination of adoptive CIK cells therapy with rh-endostatin antiangiogenic therapy to test if any synergistic antitumor responses would be obtained from this combination. Endostatin has been demonstrated of biphasic dose-response characteristic and the maximum antitumor activity is obtained within a narrow range of protein concentration. The therapeutic activity of endostatin is decreased above and below this concentration [32]. In our preliminary experiments, we used three different doses of rh-endostatin, including 20 mg/kg, 10 mg/kg and 5 mg/kg in the treatment of A549 lung cancer bearing mice and did not find any significant difference in the antitumor effect among the three doses. Thus, we chose a low dose of 5 mg/kg rh-endostatin daily for 7 days in the present study.

In human A549 and SPC-A1, and murine Lewis lung carcinoma xenografts, rh-endostatin and CIK cells therapy alone had little or no impact on tumor growth. The combination therapy was more effective than either therapy utilized alone and showed significant inhibition on tumor growth. In these experiments, adoptive CIK cells transfer alone did not cause significant tumor growth inhibition. There are several potentials factors according for the absence of efficiency: firstly, the non-small lung cancer cells we used as targets were insensitive to CIK cell-mediated lysis. Secondly, the efficacy of CIK cells adoptive therapy was closely related with the number of transfused CIK cells. In our experiments, mice were given two doses of CIK cells (2×10^7^ cells per dose), which may be insufficient to induce effective antitumor immune response. Thirdly, abnormal tumor vasculature and immunosuppressive tumor microenvironment impede the infiltration of CIK cells into tumor tissue and limit the activity of CIK cells. In the present experiment, the transfused CIK cells may be unable to migrate into the tumor parenchyma and therefore cannot exert a distinguished antitumor effect. In addition, the resistance of lung cancer to adoptive immunotherapy has been attributed to tumor-associated immune suppression, due in part to hypoxia in the tumor microenvironment [33], [34]. In our study, we found that rh-endostatin monotherapy did not exert obvious antitumor effect. The possible mechanism is that angiogenesis in the tumor may be driven by additional factors, such as fibroblast growth factor 1 (FGF-1), FGF-2, transforming growth factor-β (TGF-β), platelet-derived endothelial cell growth factor (PD-ECGF), and placental growth factor (PIGF) [35]. As a result, tumors may escape antiangiogenesis therapy by exploiting alternative angiogenic factors to generate neo-vasculature.

To further investigate the underlying mechanisms for the synergic antitumor effect observed by combination therapy, we examined the effect of rh-endostatin on tumor vasculature. We found that rh-endostatin normalized tumor vasculature by decreasing microvascular density and improving pericyte coverage which is a “normalization marker”. As we know, in physiologic condition, the vasculature is relatively permeable for the bidirectional exchange of small molecules (gases, nutrients and waste products), and to a lesser extent, of plasma proteins within the tissues. However, in pathological conditions such as cancer, vascular permeability to macromolecules is increased which induces high interstitial fluid pressure and poor perfusion, impeding the delivery of oxygen and drugs [36]. Gd-DTPA is a low-molecular-weight paramagnetic contrast (0.55 kD). Gd-DTPA-based DCE-MRI may be a useful non-invasive imaging strategy for characterizing the physiological microenvironment of tumors. It has been reported that tumors with low K^trans^ values were resistant to radiation therapy and had higher fractions of radiation resistant hypoxic cells. Recently, it is reported that compared with poorly metastatic tumors, highly metastatic tumors showed lower values of K^trans^ and K^trans^ for Gd-DTPA was reversely correlated to interstitial fluid pressure [37], [38]. In our present study, K^trans^ for Gd-DTPA was analyzed to test the vascular permeability to small molecules and K^trans^ was significantly increased by rh-endostatin treatment. Increased K^trans^ indicated increased tumor vascular permeability to small molecules, prognosticating a higher penetration for drugs and oxygen and a decreased interstitial fluid pressure in the tumor microenvironment. Intravital microscope was used to test the extravasation of Evans Blue into the tumor. Evans blue extravasation has been widely used in detecting tumor vascular permeability. It has been reported that a reduction in blood vessel permeability to Evans blue indicated a reduction in the aberrant tumor blood vessel hyperpermeability which is correlated with compromised blood flow, increased interstitial fluid pressure and consequently impaired drug delivery. Thus the reduction in extravasation of Evans blue was interpreted as a sign of blood vessel normalization. Our data showed that the extravasation of Evans blue was significantly delayed which probably predicting a reduced tumor hyperpermeability and decreased interstitial fluid pressure. Tumor oxygenation was detected by pimonidazole staining and our results showed that tumor hypoxia was significantly decreased after the administration of rh-endostatin. Continuous section slides were made and stained for hypoxia and tumor infiltrating CIK cells respectively. We found that less CIK cells accumulated in hypoxic area compared with normoxic area, indicating impaired CIK cells recruitment to tumor tissue by hypoxia. From the data above, we could know that the administration of rh-endostatin could normalize tumor vasculature and modulate hypoxic tumor microenvironment.

It has been previously shown that hypoxia suppressed the antitumor immune effector cells and enhanced the ability of tumors to escape from immunosurveillance [39], [40]. Hypoxia decreases the sensitivity of tumor cells to lymphocyte-mediated killing and impairs dendritic cell maturation which displays poor T cell stimulatory activity [39], [41]. We next designed in vitro experiments which mimicked the in vivo hypoxic tumor microenvironment to test whether hypoxia affect the proliferation, migration and cytotoxicity of CIK cells. We found that the proliferation, cytotoxicity of CIK cells were inhibited by low oxygen level, which were consistent with a previous report which suggested that hypoxia impaired T cell growth, survival and cytotoxicity [42]. Our findings also showed that hypoxia reduced the migration of CIK cells in vitro. We thus investigated whether the combination treatment could have an impact on the infiltration of transferred CIK cells into the tumor and spleen of A549 tumor-bearing mice. In our study, increased percentages of transferred CIK cells infiltration into the tumor and spleen of tumor-bearing mice were found after the administration of rh-endostatin. In the murine Lewis lung carcinoma model, we found that the combination therapy was much more efficient than either protocol alone in terms of accumulation of intratumoral CD3^+^ T lymphocytes. In addition, combination therapy induced more tumor necrosis compared with monotherapy or control group. Our study clearly showed a relationship between enhanced tumor growth retardation with improved lymphocytes infiltration.

Our findings demonstrated that rh-endostatin significantly reduced the hypoxic area in tumor microenvironment and increased the migration of CIK cells into tumor tissues. Thus it is possible that hypoxia is the main cause that inhibits the infiltration of lymphocytes into tumor parenchyma. Hypoxia has been reported to affect gene expression associated with increased angiogenesis, resulting in increased VEGF and receptor levels [43]. Because of exposure to angiogenic factors like VEGFs and FGFs in tumor microenvironment, tumor endothelial cells down-regulate the expression of intercellular cell adhesion molecule-1/2 (ICAM-1/2), vascular endothelial cell adhesion molecule-1 (VCAM-1) and CD34 which are related with leukocyte-vessel wall interactions [44], [45]. From the information above, we speculate that hypoxia up-regulates the expression of VEGF and the latter decreases the expression of endothelial cell adhesion molecules, resulting in impaired infiltration of lymphocytes into tumor parenchyma. We tested the effect of hypoxia on CIK cells adhesion to HUVECs and the adhesion molecules, ICAM-1 and VCAM-1 expression level by HUVECs. It showed that hypoxia impeded the adhesion ability of CIK cells to HUVECs by decreasing the expression of ICAM-1 and VCAM-1 by HUVECs. Continuous section slides were stained for ICAM-1, VCAM-1 and CIK cells respectively to figure out the spacial relationship between CIK cells infiltration and the endothelial cell adhesion molecules within the tumor. The accumulation of CIK cells was increased around the tumor vessels with high expressions of ICAM-1 and VCAM-1, indicating that CIK cells infiltration is related with endothelial cell adhesion molecules.

Tumor-associated immunosuppression, as a key role in tumor evasion of immune system, can be modulated by factors secreted by the tumor or the tumor microenvironment or by suppressive immune effector cells. In the present study, we focused on two important types of suppressive immune effector cells: MDSCs and TAMs. The correlation between abnormal accumulation of MDSCs and tumor progression has been observed in both preclinical and clinical researches [46], [47]. MDSCs exhibit potent immunosuppressive activity and inhibit innate and adoptive immunity [48]. Our research showed that after the delivery of rh-endostatin, the percentage of MDSCs in the tumor was significantly reduced and the pearson correlation analysis suggested an correlation between decreased intratumoral hypoxia with reduced MDSCs accumulation. Based on the aforementioned studies, we presumed that the reduced accumulation of MDSCs provided a better ground for the development of immune response. Tumor-associated macrophages, as an important component in the tumor stroma, are able to accelerate tumor growth by promoting angiogenesis, inducing immune suppression, and enhancing tumor invasion and metastasis [49], [50]. Macrophages adopt different phenotypes depending on tumor microenvironment and are generally divided into M1 and M2 phenotypes [51]. TAMs have been reported to display an M2-phenotype. It has been reported that M2-phenotype is associated with MHC II^low^ subset while M1-phenotype is associated with MHC II^high^ subset [52], [53]. Thus in the present study, we use MHC class II molecule to distinguish M1 and M2 macrophages. However, we did not find any significant difference in the infiltration of TAMs after the administration of rh-endostatin, which is probably due to the markers for the identification of TAMs are not specific and accurate enough. In the future research, more work will be focused on the identification of TAMs subsets and the role of TAMs in tumor microenvironment.

### Conclusion

Our studies provided primary evidence that rh-endostatin enhanced the antitumor activity of adoptively transferred CIK cells, and the synergistic antitumor effect was probably related to the normalized tumor vasculature and reduced hypoxic tumor microenvironment. In vitro hypoxic culture condition inhibited the proliferation, migration, adhesion and cytotoxicity of CIK cells. Rh-endostatin improved the infiltration of adoptive transferred CIK cells into the tumor and spleen and decreased the accumulation of intratumoral suppressive effector, which providing a better immune microenvironment for transferred CIK cells. Our studies reveal novel mechanisms by which rh-endostatin enhances the antitumor activity of CIK cells and may provide a rational basis for combining antiangiogenesis and immunotherapy in curing advanced stage lung cancer patients.

## Supporting Information

Figure S1 **Treatment Schematic.** BALB/C nude mice were challenged subcutaneously in the right flank with 100 µl (1×10^7^/ml) A549 cells and were treated with the respective regimens according to the treatment schematic. The day when treatment started was designated d0. The antiangiogenesis therapy in this study was subcutaneous injection of 5 mg/kg rh-endostatin for 7 days and the adoptive immunotherapy consisted of two intravenous transfusion of CIK cells at d6 and d9 (2×10^7^ cells per dose in a total volume of 100 µl). Group NS, treated with normal saline. Group EN, treated with rh-endostatin alone. Group CIK, treated with CIK cells alone. Group EN+CIK, treated with rh-endostatin followed by transfusion of CIK cells. The experiment was repeated with four groups of C57B/6 mice carrying Lewis lung carcinoma and four groups of BALB/C nude mice bearing SPC-A1 lung carcinoma.
(TIF)

Figure S2 **Characterization of phenotypes of CIK cells.** In order to characterize the phenotypes of CIK cells, cells cultured for 7, 14 and 21 days were harvested and stained for 30 min at 4°C with the following FITC or PE-conjugated monoclonal antibodies (mAbs): anti-CD3 and anti-CD56. By flow cytometry, the expression of surface markers, CD3, CD56 were examined and recorded. There are two main subpopulations of CIK cells, one expressing both the CD3 and CD56 molecules (CD3^+^CD56^+^) and the other presenting a CD3^+^CD56^−^ phenotype. After incubated for 7, 14 and 21 days, phenotypes of CIK cells were detected.
(TIF)

Figure S3 **Tumor vascular permeability assessed by intravital microscopy.** A549 tumor-bearing mice were treated with rh-endostatin (5 mg/kg, s.c.) for consecutive 7 days with normal saline as control. On days 3, 6 and 9, intravital microscopy were performed to test Evans blue extravasation. A, exposure of tumor surface and intravenous injection of Evans blue in to BALB/c mice. B, the equipment for intravital microscopy. C, representative figure of Evans blue infused tumor vessels at 100× magnification. D, representative figure of clearly shown tumor vessels as indicated by green arrows at 100× magnification. E, representative figure of blurry tumor vessels as indicated by green arrows at 100× magnification.
(TIF)

Figure S4 **Hypoxia inhibits the accumulation of CIK cells into tumor tissue in vivo.** A549 tumor-bearing mice were transfused i.v. with CIK cells. Twenty four hours after CIK cells transfusion, mice were given pimonidazole and mice were sacrificed 4 hours later. Continuous sections slides were made and tumor hypoxia and tumor infiltrating CIK cells were analyzed respectively. Tumor hypoxic areas were stained by monoclonal antibody (Mab1) against protein adducts of pimonidazole. Tumor infiltrating CIK cells were stained by anti-CD3 antibodies. A, representative figure of tumor hypoxic area at 100× magnification. B, representative figure of tumor infiltrating CD3^+^ CIK cells at 100× magnification. C, representative image showing tumor infiltrating CD3^+^ CIK cells in normoxic tumor area as indicated by black arrow at 400× magnification. D and E, images showing tumor infiltrating CD3^+^ CIK cells in hypoxic tumor area as indicated by black arrow at 400× magnification.
(TIF)

Figure S5 **Hypoxia impedes the adhesion of CIK cells to HUVECs and depresses the expression of ICAM-1 and VCAM-1 by HUVECs.** Subconfluent monolayers of HUVECs in 6-well plates were preincubated in hypoxic or normoxic culture condition for 48 h. Following preincubation, CIK cells were transferred to the HUVECs cultures and incubated for 24 h under the same culture condition as the HUVECs. Adherent CIK cells were stained by rabbit anti-mouse CD3 antibodies to detect CIK cells. Images of adherent CIK cells were acquired by using Olympus BX-60 microscope at 200× magnification. After incubation in hypoxic or normoxic culture condition for 48 h, HUVECs were stained with anti-ICAM-1 antibodies and anti-VCAM-1 antibodies. Images of adhesion molecules were acquired by using Olympus BX-60 microscope at 400× magnification.
(TIF)

Figure S6 **Rh-endostatin has no effect on the accumulation of TAMs.** C57BL/6 mice were injected s.c. with Lewis lung carcinoma cells and when tumor volume reached 100 mm^3^ treatment was initiated. After administration of rh-endostatin for consecutive 7 days, tumor-bearing mice were sacrificed and single cell suspensions of spleen, lymph node and tumor tissue were prepared to analyze TAMs frequency by flow cytometry. Bar graph depicts the percentages of TAMs in the spleen, lymph node or the tumor. Columns, mean; Bars, SE.
(TIF)
