# Supplementary figures and images for: Combining Antiangiogenic Therapy with Adoptive Cell Immunotherapy Exerts Better Antitumor Effects in Non-Small Cell Lung Cancer Models
Source: PLoS One. 2013 Jun 14;8(6):e65757. doi: 10.1371/journal.pone.0065757 (PMC3683034; doi:10.1371/journal.pone.0065757)

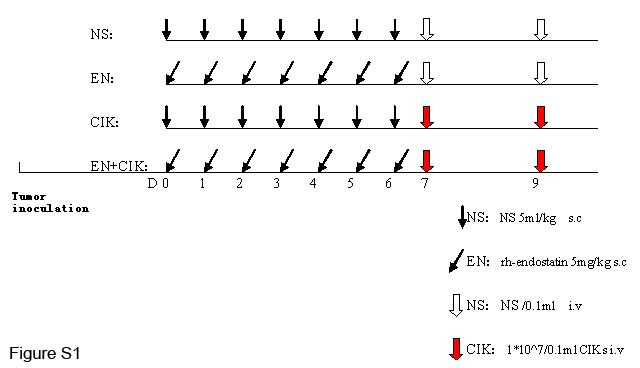

Supplement: Figure S1 — Treatment Schematic. BALB/C nude mice were challenged subcutaneously in the right flank with 100 µl (1×10^7^/ml) A549 cells and were treated with the respective regimens according to the treatment schematic. The day when treatment started was designated d0. The antiangiogenesis therapy in this study was subcutaneous injection of 5 mg/kg rh-endostatin for 7 days and the adoptive immunotherapy consisted of two intravenous transfusion of CIK cells at d6 and d9 (2×10^7^ cells per dose in a total volume of 100 µl). Group NS, treated with normal saline. Group EN, treated with rh-endostatin alone. Group CIK, treated with CIK cells alone. Group EN+CIK, treated with rh-endostatin followed by transfusion of CIK cells. The experiment was repeated with four groups of C57B/6 mice carrying Lewis lung carcinoma and four groups of BALB/C nude mice bearing SPC-A1 lung carcinoma. (TIF) [file pone.0065757.s001.tif]

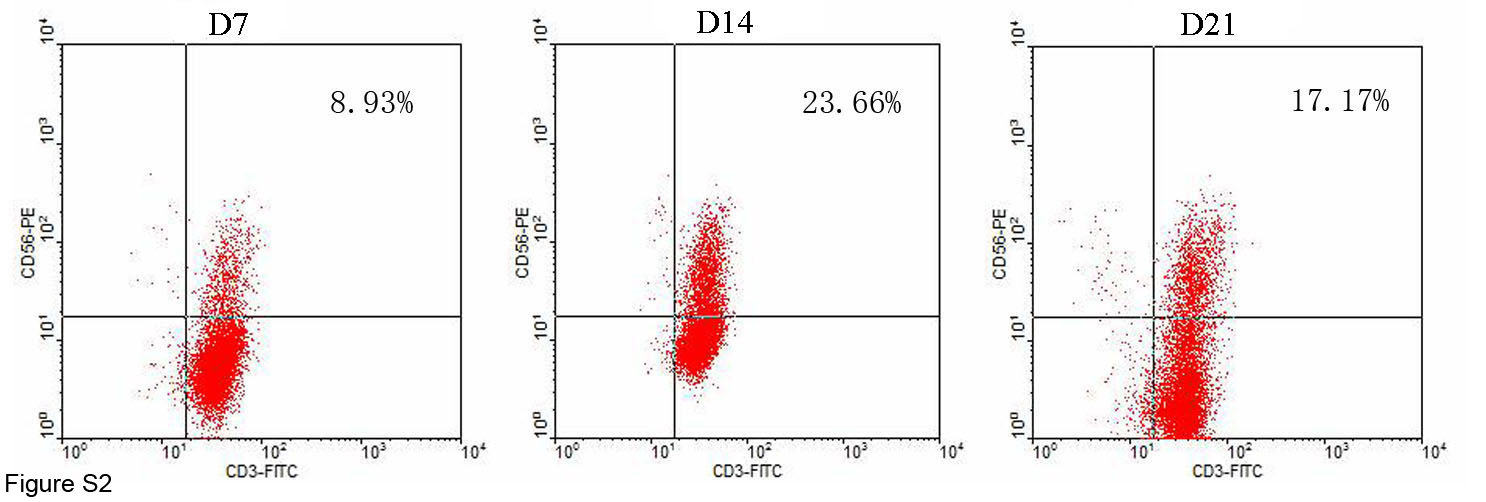

Supplement: Figure S2 — Characterization of phenotypes of CIK cells. In order to characterize the phenotypes of CIK cells, cells cultured for 7, 14 and 21 days were harvested and stained for 30 min at 4°C with the following FITC or PE-conjugated monoclonal antibodies (mAbs): anti-CD3 and anti-CD56. By flow cytometry, the expression of surface markers, CD3, CD56 were examined and recorded. There are two main subpopulations of CIK cells, one expressing both the CD3 and CD56 molecules (CD3+CD56+) and the other presenting a CD3+CD56− phenotype. After incubated for 7, 14 and 21 days, phenotypes of CIK cells were detected. (TIF) [file pone.0065757.s002.tif]

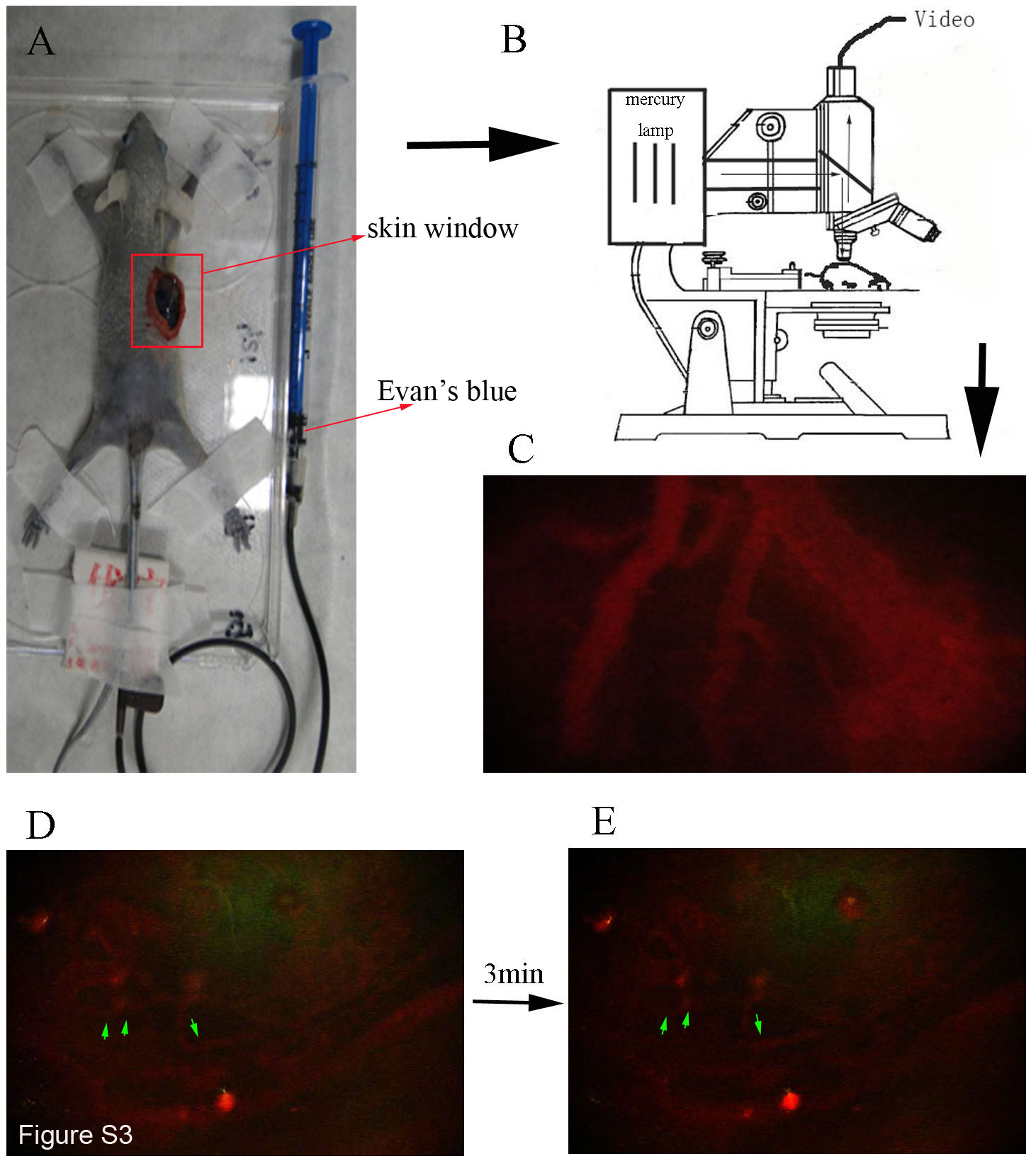

Supplement: Figure S3 — Tumor vascular permeability assessed by intravital microscopy. A549 tumor-bearing mice were treated with rh-endostatin (5 mg/kg, s.c.) for consecutive 7 days with normal saline as control. On days 3, 6 and 9, intravital microscopy were performed to test Evans blue extravasation. A, exposure of tumor surface and intravenous injection of Evans blue in to BALB/c mice. B, the equipment for intravital microscopy. C, representative figure of Evans blue infused tumor vessels at 100× magnification. D, representative figure of clearly shown tumor vessels as indicated by green arrows at 100× magnification. E, representative figure of blurry tumor vessels as indicated by green arrows at 100× magnification. (TIF) [file pone.0065757.s003.tif]

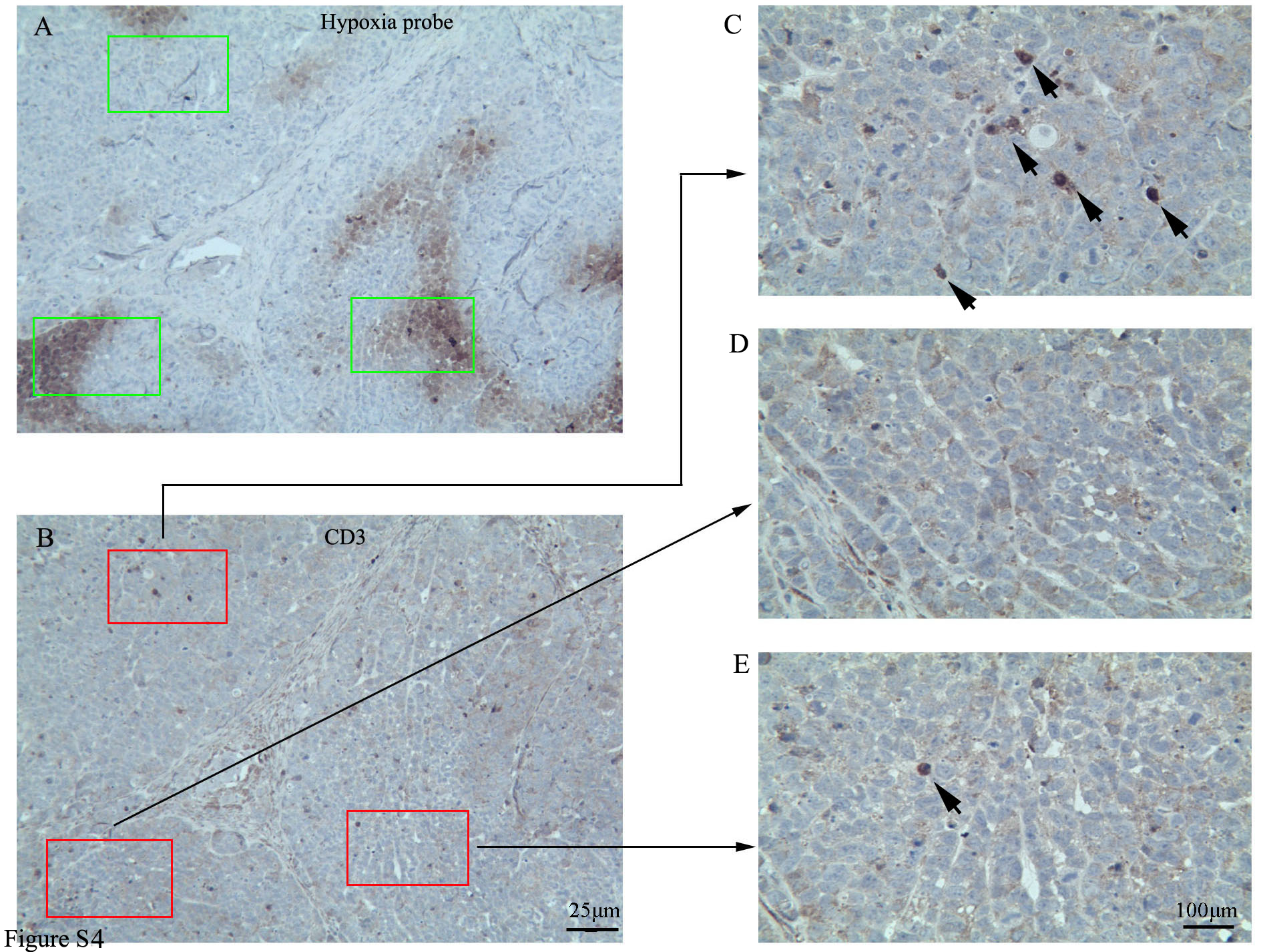

Supplement: Figure S4 — Hypoxia inhibits the accumulation of CIK cells into tumor tissue in vivo. A549 tumor-bearing mice were transfused i.v. with CIK cells. Twenty four hours after CIK cells transfusion, mice were given pimonidazole and mice were sacrificed 4 hours later. Continuous sections slides were made and tumor hypoxia and tumor infiltrating CIK cells were analyzed respectively. Tumor hypoxic areas were stained by monoclonal antibody (Mab1) against protein adducts of pimonidazole. Tumor infiltrating CIK cells were stained by anti-CD3 antibodies. A, representative figure of tumor hypoxic area at 100× magnification. B, representative figure of tumor infiltrating CD3+ CIK cells at 100× magnification. C, representative image showing tumor infiltrating CD3+ CIK cells in normoxic tumor area as indicated by black arrow at 400× magnification. D and E, images showing tumor infiltrating CD3+ CIK cells in hypoxic tumor area as indicated by black arrow at 400× magnification. (TIF) [file pone.0065757.s004.tif]

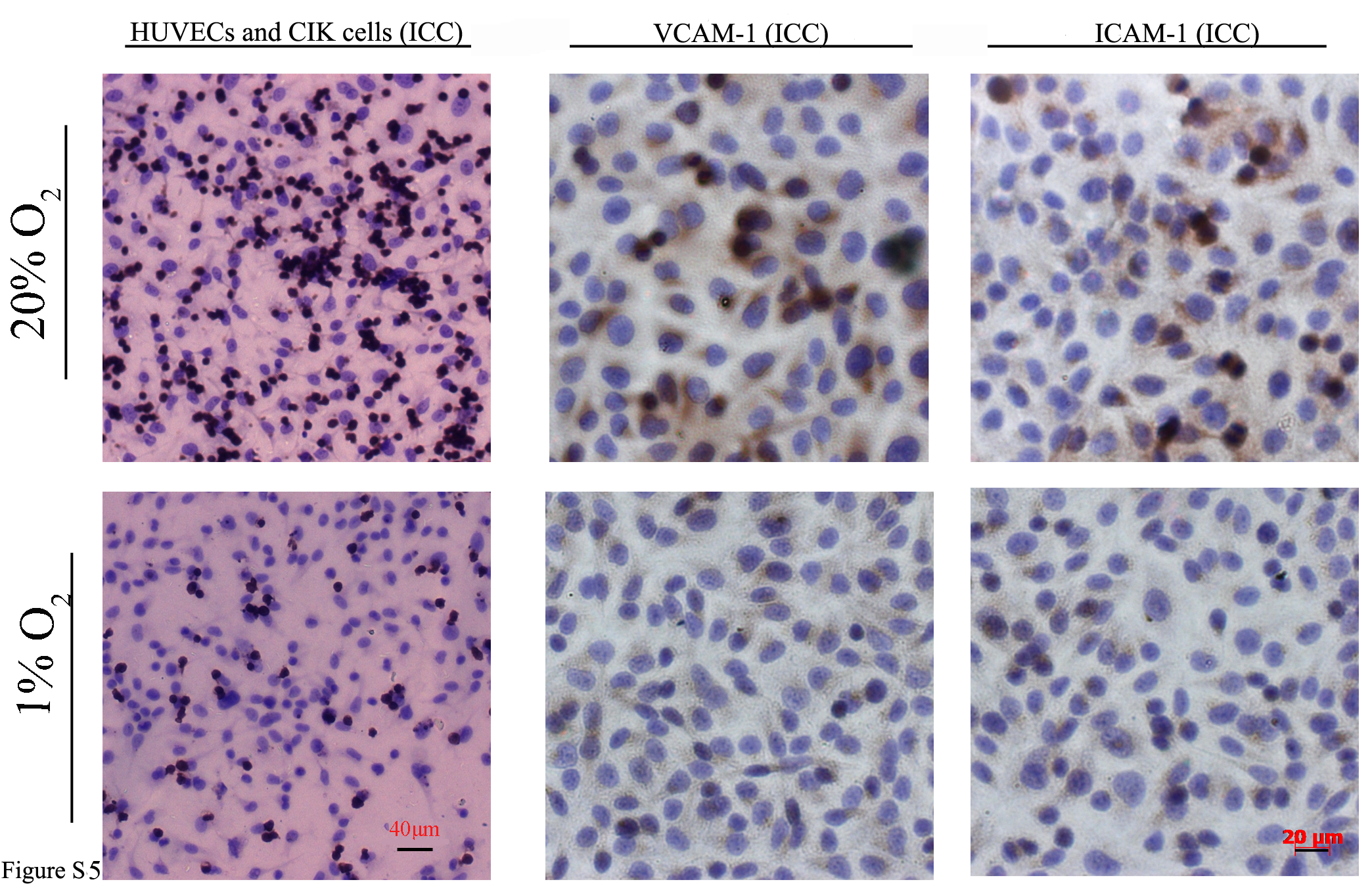

Supplement: Figure S5 — Hypoxia impedes the adhesion of CIK cells to HUVECs and depresses the expression of ICAM-1 and VCAM-1 by HUVECs. Subconfluent monolayers of HUVECs in 6-well plates were preincubated in hypoxic or normoxic culture condition for 48 h. Following preincubation, CIK cells were transferred to the HUVECs cultures and incubated for 24 h under the same culture condition as the HUVECs. Adherent CIK cells were stained by rabbit anti-mouse CD3 antibodies to detect CIK cells. Images of adherent CIK cells were acquired by using Olympus BX-60 microscope at 200× magnification. After incubation in hypoxic or normoxic culture condition for 48 h, HUVECs were stained with anti-ICAM-1 antibodies and anti-VCAM-1 antibodies. Images of adhesion molecules were acquired by using Olympus BX-60 microscope at 400× magnification. (TIF) [file pone.0065757.s005.tif]

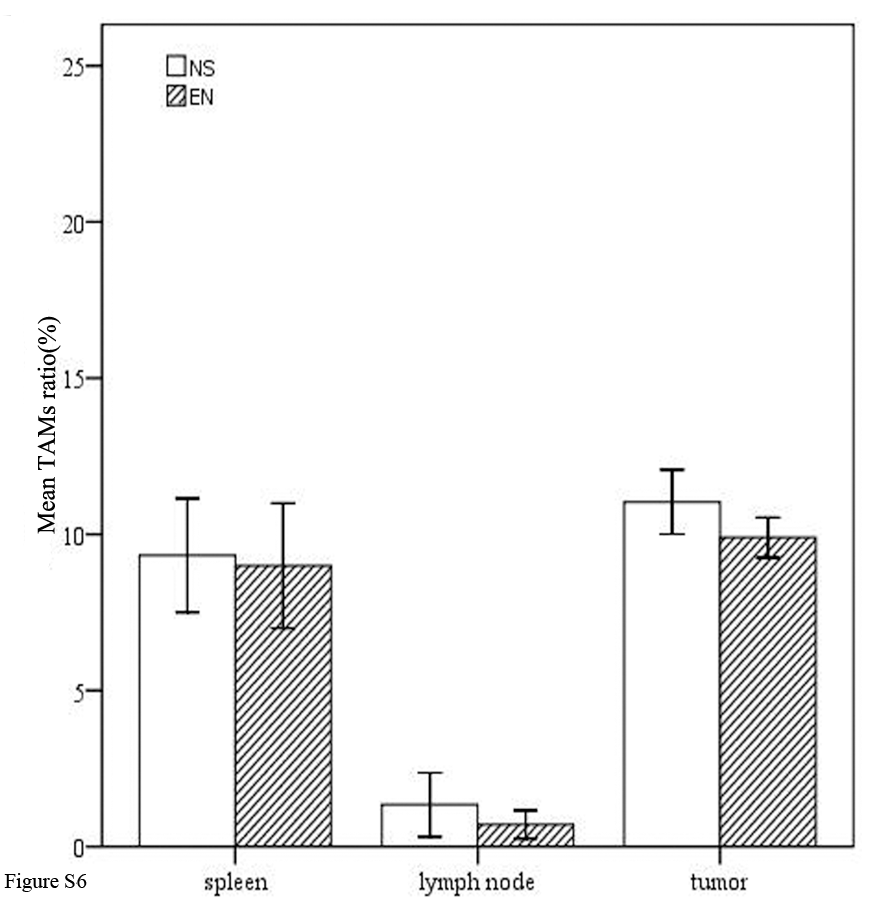

Supplement: Figure S6 — Rh-endostatin has no effect on the accumulation of TAMs. C57BL/6 mice were injected s.c. with Lewis lung carcinoma cells and when tumor volume reached 100 mm3 treatment was initiated. After administration of rh-endostatin for consecutive 7 days, tumor-bearing mice were sacrificed and single cell suspensions of spleen, lymph node and tumor tissue were prepared to analyze TAMs frequency by flow cytometry. Bar graph depicts the percentages of TAMs in the spleen, lymph node or the tumor. Columns, mean; Bars, SE. (TIF) [file pone.0065757.s006.tif]
